# Binocular indirect ophthalmo microscope-assistant gas-perfused pars plana vitrectomy: A novel technique for vitreous sample acquisition

**DOI:** 10.1097/MD.0000000000005503

**Published:** 2016-12-09

**Authors:** Luyi Zhang, Xiaoli Yang, Qingqing Zheng, Miaoqin Wu

**Affiliations:** Department of Ophthalmology, Zhejiang Provincial People's Hospital, Hangzhou, Zhejiang, China.

**Keywords:** gas-perfused, primary intraocular lymphoma, vitrectomy, vitreous sample

## Abstract

The vitreous sample has been used for the diagnosis of uveitis and intraocular malignancy for decades. The sample volume is usually limited to 1 mL with current techniques. In the present study, a novel technique for higher amount of vitreous sample acquisition, that is, Binocular Indirect Ophthalmo Microscope-assistant gas-perfused pars plana vitrectomy (BAG-PPV) was invented.

For diagnostic purpose, BAG-PPV with 23-ga vitrectomy system was performed on a 54-year-old Chinese male with the symptom of bilateral atypical uveitis. More than 3 mL of vitreous sample per eye was collected without any significant complications. Cytopathology was confirmed on the basis of cell surface markers and released cytokines by flow cytometry analysis and cytokine assays respectively.

A monoclonal B-cell population with the pattern of CD5^−^, CD10^−^, cyKi67^+^, CD71^+^, FMC7^+^, CD23^−^, and kappa light chain single expression for the right eye and a monoclonal B-cell pattern with CD5^−^, CD10^−^, cyKi67^+^, and kappa light chain restriction for the left eye were identified. The cytokine assay revealed high levels of interleukin (IL)-10 (90,838.30 and 41,098.0 pg/mL for the right and left eyes, respectively) and IL10/IL6 ratios for both eyes (with 90.78 and 63.26 for the IL10/IL6 ratios of the right and left eyes, respectively), while those for the cerebrospinal fluid were low (4.77 pg/mL for the IL10 level and 0.65 for the IL10/IL6 ratio). Based on the results, the patient was diagnosed with primary intraocular lymphoma for bilateral eyes.

Our results demonstrated that diagnostic vitrectomy with BAG-PPV using the 23-ga vitrectomy system was safe, efficient, and able to provide useful diagnostic information for suspicious intraocular malignancy and other atypical uveitis.

## Abstract 2

Supplemental Digital Content is available in the text

## 1 Introduction

The vitreous specimen obtained with pars plana vitrectomy (PPV) has been used for the diagnosis of intraocular diseases for decades.^[1]^ The collected vitreous samples are then used for further analysis with several methods, such as cytological examination, flow cytometry analysis, and polymerase chain reaction analysis. However, it is usually difficult to collect high quality of samples using standard methods due to either the small volume or the intensive dilution, which compromises the veracity of the diagnosis.

The most common pathological type of primary intraocular lymphoma (PIOL), one of the highly malignant subtypes of the primary central nervous system lymphoma is the aggressive large B cell-type.^[2–4]^ Most PIOLs are primary vitreoretinal lymphomas with low incidence counting for <1% of intraocular tumors and only 1% of non-Hodgkin lymphoma,^[5]^ plus the highly varieties of clinical manifestations-known as masquerade syndrome, and high progressiveness, so the diagnosis of PIOL is challenging. In this study, we presented a novel method BAG-PPV, the abbreviation for the Binocular Indirect Ophthalmo Microscope (BIOM)-assistant gas-perfused pars plana vitrectomy. With this technique, enough amounts of vitreous sample can be easily collected for further cytokine profiling and flow cytometry analysis, leading to more reliable diagnosis of PIOL, which was previously masqueraded as bilateral uveitis.

## 2 Methods

### 2.1 Patient

A 54-year-old Chinese male visited our department with complaint of gradual bilateral vision loss during the past more than 1 year. He had been irregularly treated for uveitis with steroid and cyclosporine A without significant efficacy. All blood tests were negative except for the slight increase of lambda light chain and kappa light chain. Pathogenic examinations were also negative with regard to *Treponema pallidum*, human immunodeficiency virus, *Mycobacterium tuberculosis*, *Cytomegalovirus*, Epstein–Barr virus, and herpes virus. Serologic markers of autoimmune diseases, including antinuclear antibodies, rheumatoid factor, and anti-double-stranded deoxyribonucleic acid antibodies, were negative. The patient also underwent an enhanced brain magnetic resonance imaging, which revealed multiple gyriform enhancements of bilateral frontal, parietal, temporal, occipital lobes, and corpus callosum, suggesting inflammatory lesions. The cerebrospinal fluid analysis demonstrated a slight increase of protein content (50.40 mg/dL). Flow cytometry analysis and cytokine assay also presented negative results. The ocular examination revealed visual acuity of light perception for both eyes. For the right eye, slit-lamp examination detected cells in the anterior chamber and the keratic precipitates. Vitreous opacity, retinal hemorrhage, yellowish-white retinal, and subretinal infiltrations were also found at the posterior region. For the left eye, the anterior chamber cells were detected, while in the posterior segment, mild vitreous opacity, diffuse nummularis retinal pigment epithelium change, and multifocal yellowish-white butyrous subretinal infiltrations above the optic disc were also found.

The patient showed no obvious evidence for infection or autoimmune diseases and had negative response to steroid and immunosuppressive agents, which led us to consider the potential diagnosis of masquerade syndrome. Based on these, BAG-PPV was performed for the purpose of diagnosis and therapy.

The patient was fully informed of the potential risks and all possible postoperative consequences before the surgery. The written informed consents were obtained from the patient's family member after the discussion of the procedure. This study was consistent with the Declaration of Helsinki and was approved by the ethics committee of Zhejiang Provincial People's Hospital before applying this surgical procedure clinically.

### 2.2 Surgical technique

**Figure 1:**
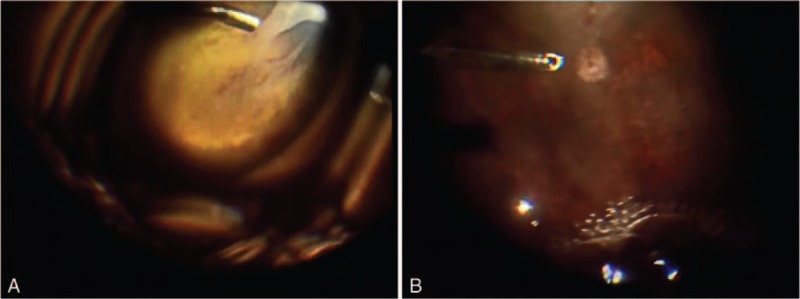
Pictures during the surgery. (A) The right eye and (B) the left eye. (We provide the video for the surgery on-line https://spaces.hightail.com/space/lBZDi.)

Before surgery, pupils of the patient were sufficiently dilated with tropicamide phenylephrine eye drops (Mydrin-p, Santen Oy., Tampere, Finland). The vitrectomies were performed under retrobulbar anesthesia with injections of 2% lidocaine (Lidocain Hydrochloride Injection, Shanghai Harvest Pharmaceutical CO., Shanghai, China) and 0.75% bupivacaine (Bupivacaine Hydrochloride Injection, Shanghai Harvest Pharmaceutical CO., Shanghai, China). BAG-PPV was performed using 3 suture-less 23-ga sclerotomies in the inferotemporal, superotemporal, and superonasal quadrants 3.5 mm from the limbus. The infusion cannula was inserted into the inferotemporal cannula perfused by aseptic air. The suction pipe of the vitrectomy cutter was connected to a 5-mL aseptic syringe. The noncontact wide-angle viewing system (BIOM, Oculus Inc., Wetzlar, Germany) was used for visualization. First, the core vitrectomy was performed with vacuum aspiration of 280-mm Hg, cutting rate of 6000/min, and 35-mm Hg air pressure (MEGATRON S4, Geuder AC, Heidelberg, Germany), with the assistant pumping the syringe at the same time. The peripheral vitrectomy was required for additional sample only if the volume was not enough. It was noteworthy that the surgeon and the assistant should cut and pump cooperatively so as to maintain suitable intraocular pressure and effective specimen collection (Fig. 1). The undiluted vitreous sample was collected and immediately sent for cytokine assessment, flow cytometry analysis, and microbiological and pathological examinations. For the right eye, we applied perfusate into the infusion cannula instead of air and performed the standard vitrectomy combined with intraocular laser coagulation and injection of silicone oil due to the progression of the disease (Video, Supplemental video, which demonstrates the surgery of the right eye). For the left eye, we simply performed the diagnostic vitrectomy since it was in a possibly quiescent period.

## 3 Results

The BAG-PPV was performed for the bilateral eyes of the patient by the same surgeon (M W) in sequence. Eventually, 3.5 mL (from the right eye, sample A) and 3 mL (from the left eye, sample B) of vitreous samples were collected. The patient then received intraocular injection of methotrexate. The methotrexate (400 μg) was injected to the affected eyes twice a week during the initial 4 weeks and once a week for the subsequent 8 weeks, and then adjusted to monthly for 9 months.

### 3.1 Flow cytometry analysis of the vitreous samples

**Figure 2:**
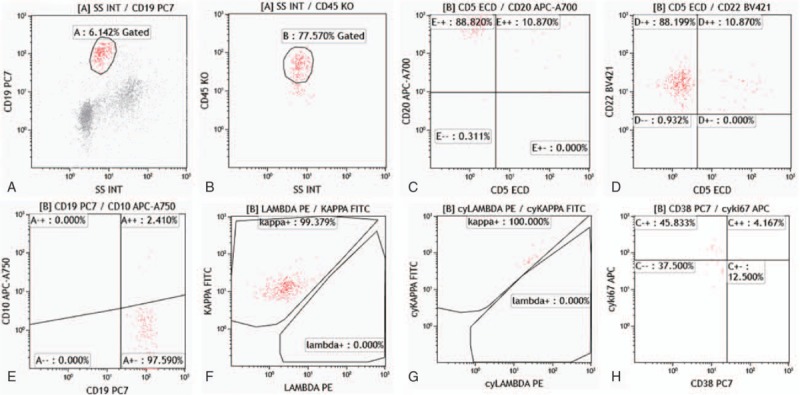
Identification of the abnormal B-cell population from the right eye sample on the basis of their forward- and side scatter characteristics. (A and B) The plot showed, in red, the abnormal monoclonal B-cell population which were CD19 and CD45 positive; (C–E) the plot showed, in red, the cells that were CD5 and CD10 negative, CD20 and CD22 positive; (F and G) the cells showed the sole expression of the Kappa light chain; and (H) CyKi67 positive, indicating the abnormal proliferation of cells.

**Figure 3:**
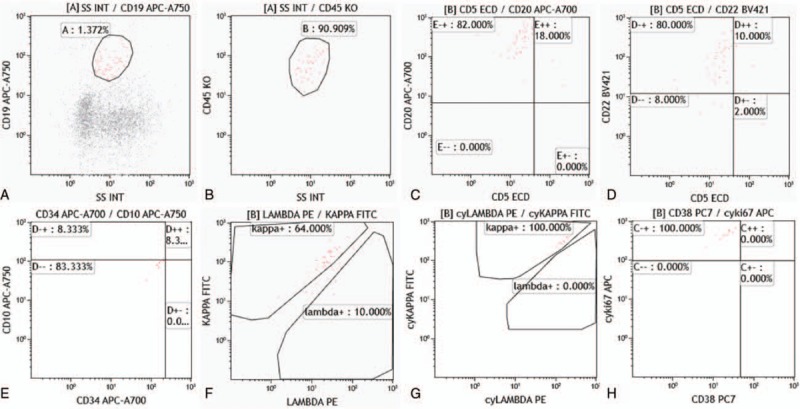
Identification of the abnormal B-cell population from the left eye sample on the basis of their forward- and side scatter characteristics. (A and B) The plot showed, in red, the abnormal monoclonal B-cell population which were CD19 and CD45 positive; (C–E) the plot showed, in red, the cells were CD5 and CD10 negative, CD22 and CD20 positive; (F and G) the cells showed Kappa light chain restriction; and (H) CyKi67 positive, indicating the abnormal proliferation of cells.

Flow cytometry analysis detected over 3400 cells in sample A. The assay defined an abnormal monoclonal B-cell population with the patterns as positive for CD45, CD19, CD20, CD22, CD71, HLA-DR, FMC7, sIgM, and cyKi67, and negative for CD10, CD5, CD7, CD34, CD117, CD33, CD13, CD34, CD103, CD11c, CD25, CD23, CD27, CD28, CD138, CD30, CD123, CD38, with kappa light chain single expression (Fig. 2), which accounted for 4.76% of the total cells by double gates selection of CD19 and CD45. In sample B, an abnormal B monoclonal cell population counting for 1.25% of the total 4009 detected cells. These cells were positive for CD45, CD19, CD20, CD22, CD71, CD28, FMC7, sIgM, and cyKi67, and negative for CD5, CD10, CD103, CD11c, CD25, CD23, CD27, CD138, CD30, CD123, CD34, CD38, with kappa light chain restriction (Fig. 3). Our results revealed typical characteristics of diffuse large B-cell lymphoma (DLBCL).

### 3.2 Cytokine assay of the vitreous samples

The cytokine assay for the vitreous samples revealed extremely high levels of interleukin (IL)-10 in both eyes (Table 1). The ratios of IL-10/IL-6 were much higher than 1.0 as shown as 90.78 for the right eye and 63.26 for the left. These results provide another evidence as DLBCL.

**Table 1:**
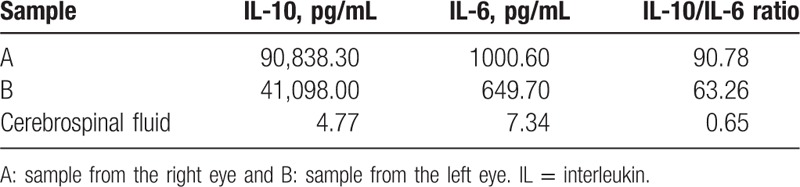
Cytokine assay results of IL-10 and IL-6.

### 3.3 Cytological and microbiological examinations

Cytological test was only performed for the left eye and showed negative result. It is probably due to poor cell viability and small cell quantity in the sample. Moreover, the microbiological examinations for both eyes were negative as well, which excluded the possibility of infective endophthalmitis.

### 3.4 Postoperative eye conditions

**Figure 4:**
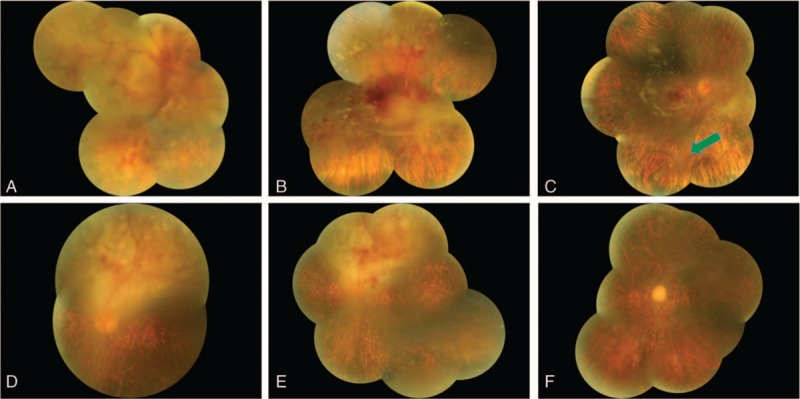
Fundus photographs in color for the right (A–C) and the left eye (D–F). (A) Preoperation, vitreous opacity, retinal hemorrhage, yellowish-white retinal and subretinal infiltrations, and retinal edema were found; (B) 2 weeks after the operation; (C) 5 weeks after the operation, the intraocular injection for 9 times, the green arrow showed the methotrexate drop. (D) Preoperation, mild vitreous opacity, diffuse nummularis retinal pigment epithelium change, and multifocal yellowish-white butyrous subretinal infiltrations with hemorrhage above the disc were found; (E) 2 weeks after the operation; (F) 5 weeks after the operation, the intraocular injection for 9 times, the infiltrations obviously decreased.

No significant complications such as retinal detachment, hemorrhage, hypotony, and hypertony related to BAG-PPV were reported during the follow-up period (5 weeks). The fundus of pre- and postoperation were shown (Fig. 4). The visual acuity remained unchanged after the surgery.

### 3.5 Outcome

Up to date, the follow-up period for the patient was already 5 weeks. The patient received bilateral intraocular methotrexate injection at a dose of 400 μg twice a week for the first 4 weeks and once for 1 week. Compared to the previous conditions (Fig. 4), the symptoms of retinal edema, lesion, hemorrhage, and infiltrations disappeared; however, the visual acuity remained the same. Further follow-up studies are required.

## 4 Discussion

The vitreous humor is the transparent, jelly-like substance that fills the middle of the eye with the volume of about 4.5 mL^[6]^ and can be used for diagnosis of atypical uveitis, suspected endophthalmitis, and other intraocular malignancies.

The intraocular specimen can be obtained by 2 major methods: needle aspiration biopsy and vitrectomy. Fine-needle aspiration biopsy is the safe procedure especially suitable for cases with mass,^[7]^ but usually complicated with intravitreal hemorrhage.^[8]^ However, due to the limited volume of the collected sample, it is not appropriate for cases with tiny or no mass at all. While vitrectomy has become the most powerful therapeutic and diagnostic tool for fundus diseases since its first introduction 40 years ago.^[9–12]^ The amount of undiluted vitreous sample that can be obtained safely is believed to be limited to 1 mL.^[13]^ However, this amount of sample acquired is thus far from enough in certain cases, especially for further diagnosis as flow cytometry analysis, microorganism examination, cytokine assays, and molecular analysis, even though the complete core vitrectomy is performed.^[14]^ Vitrectomy for the undiluted sample may also cause many issues as vitrectomy without infusion might cause lens and retinal lesions inadvertently due to poor surgical vision and might increase the risk of postoperative complications. Moreover, hypotony and negative-pressure condition during the vitrectomy without infusion could lead to choroidal and retinal detachment, hemorrhage, and edema. For the purpose to avoid the circumstances mentioned above, a dilution method is suggested, in which diluted specimen are acquired by vitrectomy with infusion. However, the following centrifuge process for collection of cells in vitreous wash fluid might damage or even destroy the cells. Furthermore, the perfusate makes the targeted cells even rarer. Besides, extra steps introduced during the collection of the diluted specimen increase the risks of microbial contamination of the sample. Therefore, the above-considered reasons make the tests of the diluted vitreous sample unreliable, especially for patients with suspected endophthalmitis. In summary, the current vitrectomy methods, either collecting undiluted samples or diluted specimen with the use of perfusate fluid, have big drawbacks and need to be improved.

In line with the actual demands, we invented BAG-PPV. Its main feature is for collecting more than 3 mL undiluted and easy-preserved vitreous fluid in a relatively short time and promote the results of the physiological and pathological examinations more authentic and accurate. In addition, compared to previous methods, it is much easier to maintain the intraocular pressure during the procedure and help to reduce the complications caused by intraoperative hypotony. Vitrecotmy under air could also significantly reduce the rate of iatrogenic retinal break formation compared to vitrecotmy with perfusate as reported in a previous study.^[15]^

Another advantage of our BAG-PPV procedure is using BIOM viewing system. The BIOM is a noncontact lens-type wide-angle viewing system with a good wide view up to 120–130 degrees, compared to the traditional one. It helps surgeon to view more peripheral areas of the retina by slightly rotating eyes without the aid from an assistant. It also has a better visibility of the fundus even in patients with small pupils, corneal opacity, or intraocular lens especially multifocal or toric intraocular lens.^[16,17]^ More importantly, the surgeon can get a wide, excellent visibility of the fundus during the gas–fluid exchange period.^[18]^ Thus a more flexible operative technique-BAG-PPV is presented with the requisition of BIOM system for a clear vision under the gas, which provides a better surgical vision than the double concave contact lens.

PIOL has been reported to have a very low rate of occurrence with high malignancy,^[5]^ and its diagnosis is considered to be arduous. The patients usually have atypical symptoms, and the acquisition and preservation of vitreous specimens are technically challenging.^[19]^ Besides, vitreous specimens do not always contain neoplastic cells, and this is especially true if there is minimal vitreous involvement by the lymphoid cells.^[20]^ Up to date, a number of tests, including flow cytometry analysis, molecular detection of clonal gene rearrangements, and cytokine profiling of intraocular fluid, have been developed for diagnosis of PIOL.^[19]^ For cytokine assay, an IL-10/IL-6 ratio greater than 1.0 was considered to be useful as the diagnostic criterion.^[21]^ In our case, the specimens from both eyes revealed an abnormal monoclonal B-cell population comprising 4.76% (the right eye) and 1.25% (the left eye) of the total cells. These cells had positive staining for CD45, CD19, CD20, CD22, and CyKi67, with kappa light chain single expression or restriction, while CD5 and CD10 were negative. Cytokine assay of these specimens revealed dramatically elevated IL10/IL6 ratios, much higher than 1.0, which led to the definite diagnosis of bilateral PIOL.

Unfortunately, we were not thoughtful enough to take the vitreous sample from the right eye for cytological examination. The left eye was at a symptomatic relief period for weeks and then the infiltration above the optic disc increased. Considering the results of flow cytometry analysis from the right eye, we performed the vitrectomy and the cytological examination for the left eye, which turned out to be false-negative partially due to the poor cell viability and small cell quantity in the sample. In addition, the left eye was relatively stable without a solid tumor; vitreous opacity was mild while the vitreous body contained a small amount of lymphocytes and other cells. Of note, the sensitivity of the cytological test is lower than flow cytometry analysis and cytokine assay.^[22]^ All the conditions described above might be the reasons leading to the negative result for the cytological examination of the left eye.

In this study, we only demonstrated BAG-PPV work efficiently on the bilateral eyes from 1 patient since the occurrence rate of PIOL is extremely low. Although BAG-PPV with 23-ga vitrectomy system might be a better alternative for current vitreous sample collection in cases of intraocular malignancy, atypical uveitis either infectious or inflammatory, and suspected endophthalmitis, more cases should be collected for further investigations on the safety in a longer postoperative period, which will be beneficial to the accurate diagnosis of intraocular malignancies in the long run.

## Acknowledgments

We thank Wanmao Ni, Qi Zhang for assisting in preparation of this manuscript.

## Supplementary Material

Supplemental Digital Content

## References

[1] Pslexas GN Green WR Goldberg MF Diagnostic pars plana vitrectomy report of a 21-year retrospective study. Trans Am Ophthalmol Soc 1995;93:281–308. 8719683 PMC1312062

[2] Chan CC Rubenstein JL Coupland SE Primary vitreoretinal lymphoma: a report from an international primary central nervous system lymphoma collaborative group symposium. Oncologist 2011;16:1589–99. 22045784 10.1634/theoncologist.2011-0210 PMC3233294

[3] Coupland SE Damato B Understanding intraocular lymphomas. Clin Experiment Ophthalmol 2008;36:564–78. 18954321 10.1111/j.1442-9071.2008.01843.x

[4] Coupland SE Foss HD Hidayat AA Extranodal marginal zone B cell lymphomas of the uvea: an analysis of 13 cases. J Pathol 2002;197:333–40. 12115879 10.1002/path.1130

[5] Bardenstein DS Intraocular lymphoma. Cancer Control 1998;5:317–25. 10761081 10.1177/107327489800500403

[6] Camille B Alain D Martine P Cytopathology of vitreous humor samples in routine practice. Acta Cytologica 2016;60:65–73. 26986556 10.1159/000444576

[7] Arun DS Carlos AM Nakul S Fine-needle aspiration biopsy of uveal melanoma: outcomes and complications. Br J Ophthalmol 2016;100:456–62. 26231747 10.1136/bjophthalmol-2015-306921

[8] Alexandre S Laurence D Raymond B Fine needle aspiration biopsy in uveal melanoma: technique, complications, and outcomes. Am J Ophthalmol 2016;162:28–34. 26556006 10.1016/j.ajo.2015.11.005

[9] Svozilkova P Heissigerova J Brichova M The role of pars plana vitrectomy in the diagnosis and treatment of uveitis. Eur J Ophthalmol 2011;21:89–97. 20853259 10.5301/ejo.2010.4040

[10] Wittenberg LA Maberley DA Ma PE Contribution of vitreous cytology to final clinical diagnosis fifteen year review of vitreous cytology specimens from one institution. Ophthalmology 2008;115:1944–50. 18672292 10.1016/j.ophtha.2008.05.022

[11] Kinoshita Y Takasu K Adashi Y Retrospective cytological study of intraocular lymphoma using vitreous and intraocular perfusion fluid. Diagn Cytopathol 2012;40:604–7. 22707324 10.1002/dc.21596

[12] Kinoshita Y Takasu K Kobayashi TK Diagnosis of intraocular lesions using vitreous humor and intraocular perfusion fluid cytology: experience with 83 cases. Diagn Cytopathol 2015;43:353–9. 25334001 10.1002/dc.23222

[13] Satoru K Kenichi N Daiju I Diagnostic efficacy of cell block method for vitreoretinal lymphoma. Diagnostic Pathology 2016;11:29. 26987877 10.1186/s13000-016-0479-1 PMC4797249

[14] Gonzales JA Chan CC Biopsy techniques and yields in diagnosing primary intraocular lymphoma. Int Ophthalmol 2007;27:241–50. 17440686 10.1007/s10792-007-9065-6 PMC2048742

[15] Michele R Stanislao R Teresio A Iatrogenic retinal breaks in 25-gauge vitrectomy under air compared with standard 25-gauge system for macular diseases. Reina 2014;34:1617–22. 10.1097/IAE.0000000000000112 24651259

[16] Inoue M Noda T Mihashi T Quality of image of grating target placed in model of human eye with corneal aberrations as observed through multifocal intraocular lenses. Am J Ophthalmol 2011;151:644–52. 21257154 10.1016/j.ajo.2010.09.029

[17] Inoue M Noda T Ohnuma K Quality of image of grating target placed in model eye and observed through toric intraocular lenses. Am J Ophthalmol 2013;155:243–52. 23036568 10.1016/j.ajo.2012.07.017

[18] Seita M Mihori K Shin Y 23-gauge vitrectomy assisted by combined endoscopy and a wide-angle viewing system for retinal detachment with severe penetrating corneal injury: a case report. Clin Ophthalmol 2011;5:1767–70. 22267909 10.2147/OPTH.S25373 PMC3258084

[19] Davis JL Intraocular lymphoma: a clinical perspective. Eye 2013;27:153–62. 23196650 10.1038/eye.2012.250 PMC3574250

[20] Raparia K Chang CC Chevez-Barrios P Intraocular lymphoma: diagnostic approach and immunophenotypic findings in vitrectomy specimens. Arch Pathol Lab Med 2009;133:1233–7. 19653716 10.5858/133.8.1233

[21] Whitcup SM Stark-Vancs V Wittes RE Association of interleukin 10 in the vitreous and cerebrospinal fluid and primary central nervous system lymphoma. Arch Ophthalmol 1997;115:1157–60. 9298057 10.1001/archopht.1997.01100160327010

[22] Kimura K Usui Y Goto H The Japanese Intraocular Lymphoma Study Group. Clinical features and diagnostic significance of the intraocular fluid of 217 patients with intraocular lymphoma. Jpn J Ophthalmol 2012;56:383–9. 22661396 10.1007/s10384-012-0150-7

